# The Viral G-Protein-Coupled Receptor Homologs M33 and US28 Promote Cardiac Dysfunction during Murine Cytomegalovirus Infection

**DOI:** 10.3390/v15030711

**Published:** 2023-03-09

**Authors:** Cassandra M. Bonavita, Timothy M. White, Joseph Francis, Helen E. Farrell, Nicholas J. Davis-Poynter, Rhonda D. Cardin

**Affiliations:** 1 Department of Pathobiological Sciences, School of Veterinary Medicine, Louisiana State University, Baton Rouge, LA 70803, USA; 2 Department of Comparative Biological Sciences, School of Veterinary Medicine, Louisiana State University, Baton Rouge, LA 70803, USA; 3 School of Chemistry and Molecular Bioscience, University of Queensland, Brisbane 4072, Australia; 4 Child Health Research Centre, University of Queensland, Brisbane 4101, Australia

**Keywords:** cytomegalovirus, HCMV, MCMV, cardiac dysfunction, viral pathogenesis, viral reactivation

## Abstract

Human cytomegalovirus (HCMV) is a ubiquitous pathogen that infects the majority of the world population and causes lifelong latent infection. HCMV has been shown to exacerbate cardiovascular diseases, including myocarditis, vascular sclerosis, and transplant vasculopathy. Recently, we have shown that murine CMV (MCMV) recapitulates the cardiovascular dysfunction observed in patients with HCMV-induced myocarditis. To understand the viral mechanisms involved in CMV-induced heart dysfunction, we further characterized cardiac function in response to MCMV and examined virally encoded G-protein-coupled receptor homologs (vGPCRs) US28 and M33 as potential factors that promote infection in the heart. We hypothesized that the CMV-encoded vGPCRs could exacerbate cardiovascular damage and dysfunction. Three viruses were used to evaluate the role of vGPCRs in cardiac dysfunction: wild-type MCMV, a M33-deficient virus (∆M33), and a virus with the M33 open reading frame (ORF) replaced with US28, an HCMV vGPCR (i.e., US28+). Our in vivo studies revealed that M33 plays a role in promoting cardiac dysfunction by increasing viral load and heart rate during acute infection. During latency, ΔM33-infected mice demonstrated reduced calcification, altered cellular gene expression, and less cardiac hypertrophy compared with wild-type MCMV-infected mice. Ex vivo viral reactivation from hearts was less efficient in ΔM33-infected animals. HCMV protein US28 expression restored the ability of the M33-deficient virus to reactivate from the heart. US28+ MCMV infection caused damage to the heart comparable with wild-type MCMV infection, suggesting that the US28 protein is sufficient to complement the function of M33 in the heart. Altogether, these data suggest a role for vGPCRs in viral pathogenesis in the heart and thus suggest that vGPCRs promote long-term cardiac damage and dysfunction.

## 1. Introduction

Human cytomegalovirus (HCMV), a betaherpesvirus, is a widespread pathogen that causes lifelong latent infection in much of the world population. While the most severe sequela result from systemic infections in immunocompromised patients and birth defects in infants, seropositivity also correlates with the exacerbation of many chronic diseases [1,2]. HCMV infection is associated with diseases of the cardiovascular system, including myocarditis, transplant vasculopathy, vascular sclerosis, and hypertension [3,4,5,6,7,8,9]. Damage to the heart is often accompanied by life-threatening cardiac dysfunction; for example, in patients with HCMV-induced myocarditis, problems such as sinus tachycardia, arrythmias, and ventricular overload can occur [3,10]. Understanding the viral mechanisms involved in cardiac dysfunction is crucial; however, because HCMV is a host-restricted pathogen that requires the use of human cell lines, research on HCMV pathogenesis in the heart is limited. To circumvent this issue, our lab and others’ labs utilize murine cytomegalovirus (MCMV) infection as a model for HCMV-associated cardiovascular diseases [11,12,13,14,15].

Similar to HCMV, MCMV infects many cell types and tissues, including the spleen, liver, and salivary glands [16,17]. In addition, MCMV shares a significant sequence homology with HCMV and recapitulates much of the pathology of HCMV-associated cardiovascular diseases [12,18]. Over the course of their coevolution with their hosts, betaherpesviruses have hijacked various eukaryotic proteins, such as GPCRs, and incorporated them into the viral genome [19]. HCMV encodes four putative vGPCRs, and of these, only US28 has been shown to bind and sequester chemokines similar to host GPCRs [20]. Similarly, MCMV encodes two putative chemokine receptor homologs, M33 and M78, of which M33 has been shown to act as a partial functional homolog of US28 [18,21].

Our recent studies have demonstrated similar cardiac dysfunction between MCMV-infected mice and HCMV-infected patients [15]. Here, we further evaluate MCMV-associated heart dysfunction. Previously, we demonstrated that MCMV establishes latency in the heart, as shown by the ex vivo reactivation of the virus [15]. Our current studies show virally encoded G-protein-coupled receptor homologs (vGPCRs) as a possible mechanism at play during the infection of the heart. Further, vGPCRs play a role in viral pathogenesis by modulating host signaling, immune evasion, and latency [21,22,23,24], and vGPCRs have also been implicated in contributing to HCMV-associated chronic diseases, including some cancers and cardiovascular diseases [1,25,26]. Thus, we focused our investigations on M33, an MCMV vGPCR, and its functional homolog in HCMV, US28 [21].

Although M33 has not been shown to bind chemokines like US28, M33 is capable of similar signaling pathway activation and is proposed to be the functional homolog of US28 [18,21,27,28]. In the vasculature, both US28 and M33 promote smooth muscle cell migration [24,29,30,31]. In renal allografts, US28 increases dissemination and facilitates the arterial remodeling of the vessels [32]. Unlike most viral proteins, US28 expression is detectable in the in vitro models of latency in THP-1 monocyte-like cells [33]. To date, M33 has not been detected during MCMV latency; however, M33-deficient viruses are deficient in ex vivo reactivation from the spleen and lungs, a defect that can be rescued by complementary substitution of the US28 ORF [34]. Given the similar contribution of HCMV US28 and MCMV M33 vGPCRs in smooth muscle physiology in vitro, we hypothesized that M33 may play a role in MCMV-mediated cardiac disease.

To evaluate the role of vGPCRs in cardiac dysfunction, we focused on three viruses: wild-type parental strain K181-Perth (KP), a M33-deficient virus (ΔM33), and a M33-deficient virus that expressed the HCMV US28 (US28+) [21,35]. Our results demonstrate a role for M33 in promoting cardiac dysfunction during acute and latent MCMV infection. Moreover, US28 functionally complements M33 since the US28+ virus induces damage and dysfunction in the heart at levels similar to the wild-type MCMV, and ex vivo reactivation from heart tissue is restored to wild-type levels.

## 2. Materials and Methods

### 2.1. Cell and Virus Preparation

NIH 3T3 fibroblast cells (Cell Line Services, Eppelheim, Germany) were grown in Dulbecco’s modified Eagle medium (DMEM) with 4.5 g/L glucose and L-glutamine without sodium pyruvate (Corning, NY, USA) supplemented with 10% FBS (Hyclone), 7.5% sodium bicarbonate, 4 mM HEPES, 2 mM L-glutamine, and 1% gentamycin [34]. Cells were incubated in 5% CO_2_ at 37 °C. Wild-type viruses used for studies were the strains K181+ Stanford (referred to in text as K181+; kindly provided by Ed Mocarski) and K181-Perth (referred to as KP). The M33-null mutant virus was ΔM33_BT2_ (referred to as ΔM33), which contains a *LacZ* insert disrupting the M33 gene locus [34,35]. A second mutant, ΔM33/US28-GFP (referred to as US28+), contains the HCMV US28 gene replacing the M33 gene locus [21]. Both mutants were constructed on the KP background. All virus stocks were prepared in NIH 3T3 cells because of the ΔM33 defect in salivary gland replication and were stored at −80 °C [34]. Virus titer (PFU/mL) was determined by plaque assay on NIH 3T3 cells.

### 2.2. In Vivo MCMV Infection

5-week-old female BALB/c mice were purchased from Jackson Laboratory (Bar Harbor, ME, USA). For MCMV infection, mice were intraperitoneally (i.p.) inoculated with 1 × 10^6^ pfu of MCMV. At 3, 7, 14, 70, and 90 days post infection (dpi), animals were sacrificed and tissues removed for subsequent analysis [34]. For ganciclovir studies, mice were treated by i.p. administration of 50 mg/kg/day ganciclovir (GCV) (Sigma Aldrich, Saint Louis, MO, USA) dissolved in PBS. Ganciclovir treatment began 1 day prior to infection and was administered twice daily for 14 days [36]. Mice were maintained under specific pathogen-free conditions at Louisiana State University School of Veterinary Medicine, Baton Rouge, LA, USA. All animal protocols were approved by the Institutional Animal Care and Use Committee at Louisiana State University.

### 2.3. In Vivo Acute Replication

Viral replication in tissues of MCMV-infected mice was evaluated at 3, 7, and 14 dpi by plaque assay on NIH 3T3 fibroblasts, as previously described [15,34]. Briefly, the hearts were collected, processed into 10% tissue homogenates (*w*/*v*) in DMEM media, and incubated on NIH 3T3 fibroblasts at 37 °C and 5% CO_2_ for 1 h. Following incubation, the supernatant was removed, and cells were overlayed with carboxymethyl cellulose, incubated for 5–6 days, and fixed and stained with Giemsa (Sigma Aldrich, Saint Louis, MO, USA). MCMV plaques were visualized using a light microscope. Virus titers were represented as Log_10_ PFU/mL tissue homogenate.

### 2.4. Viral DNA Quantification

Viral DNA load within the hearts was evaluated by quantitative PCR (qPCR) at 3, 7, and 14 dpi. From each heart, 0.1 g tissue was homogenized, and DNA was isolated using the QiaAmp DNA extraction kit (Qiagen, Hilden, Germany). The *early gene 1* (E1) was amplified by using E1-specific primers (F-5′TCGCCCATCGTTTCGAGA-3′), (R-5′TCTCGTAGGTCCACTGACGGA-3′), and probe (6FAM-ACTCGAGTCGGACGCTGCATCAGAAT-TAMRA), as previously described [15,37,38]. Viral copy number was determined using the pCB-E1 plasmid containing the MCMV *early gene 1*. E1 was cloned into a pcDNA3.1 plasmid by using the fast cloning technique [39,40]. The qPCR assay was conducted using an AB7000 machine (Applied Biosystems, Foster City, CA, USA) with the following temperature profiles: 50 °C for 2 min; 95 °C for 10 min; and 40 cycles of 95 °C for 15 s, 60 °C for 1 min, and 70 °C for 1 min.

### 2.5. Cardiac Function

At 14, 70, and 90 dpi, the hearts of uninfected and MCMV-infected mice were evaluated by echocardiography (Toshiba Aplio 80, Tokyo, Japan). Briefly, mice were sedated with 2% isoflurane via nose cone inhalation and the heart rate, left ventricular internal diameter (diastolic/systolic), and left ventricular posterior wall thickness (diastolic/systolic) were measured [15,41].

### 2.6. Ex Vivo MCMV Reactivation

At 70 and 90 dpi, mice were evaluated for viral reactivation from latency using the primary explant reactivation assay, as previously described [34]. Following echocardiography, the hearts were collected, minced, and cultured in DMEM media for 6 weeks. The supernatant was collected weekly, culture media was replaced, and the reactivation of virus was determined by the plaque assay of supernatants on NIH 3T3 fibroblasts. The data have been graphed as the percentage of animals reactivating from latency per group and PFU/mL. Supernatants were also titered to measure the quantitative differences in virus reactivation.

### 2.7. Histology

Hearts were evaluated for phenotypic changes at all time points. Following analysis by echocardiography, hearts were fixed in 10% formalin, embedded in paraffin, and stained with either Alizarin red solution (4 per group) to evaluate calcification or hematoxylin and eosin (2 per group) to evaluate immune cell infiltration. Images were visualized with Nano Zoomer Digital Pathology 2 software. Hematoxylin and eosin (H and E) slides were blindly evaluated by a pathologist at the LSU School of Veterinary Medicine. In addition, all hearts were given an estimated pathology score to compare calcification between groups, as previously described [15]. Scoring was determined on the basis of the estimated percentage of white deposit visible on the surface of the heart observed on the front and back of the tissue: 0 = no visible changes to the heart; 0.5 = hearts with no visible white deposit but altered pale appearance; 1 = 0–25% of the heart containing white deposit; 2 = 25–50% of the heart containing white deposit; 3 = 50–75% of the heart containing white deposit; and 4 = 75–100% of the heart containing white deposit.

### 2.8. Cellular Gene Expression

At 70 and 90 dpi, RNA was extracted from 0.1 g heart tissue, from 5 animals per group, using the RNeasy Mini Kit (Qiagen, Hilden, Germany). Following RNA extraction, 100 ng of cDNA was synthesized per sample using the RT2 First Strand Kit per manufacturer’s instructions (Qiagen, Hilden, Germany). Real-time PCR (RT-PCR) was conducted by using 25 primers sets specific to cellular genes of interest (Table S1). The RT-PCR reaction used RT2 SYBR Green ROX Mastermix Fast (Qiagen, Hilden, Germany), 1 nmol of primers, and water (in a 24 μL volume). Samples were run on an AB7000 machine (Applied Biosystems, Foster City, CA, USA) with the following temperature profile: 95 °C for 10 min, 40 cycles at 95 °C for 15 s, and 60 °C for 1 min. All samples were normalized to uninfected control hearts by using the cellular housekeeping gene *GAPDH*, and fold change was determined. All hearts were individually analyzed, and then group averages were determined following the determination of fold change. All primers were synthesized by Integrated DNA Technologies (IDT Inc., Coralville, IA, USA); they are listed in Supplementary Table S1.

### 2.9. Flow Cytometry

At 90 dpi, 5 hearts per group were collected for flow cytometry. Hearts were washed with PBS to remove peripheral blood, minced, and incubated in collagenase II solution (Thermo Fisher Scientific, Waltham, MA, USA) for 4 h at 37 °C. The tissue homogenate was then strained through a 40-micron cell strainer, layered on Lympholyte-M (Cedarlane Laboratories Ltd. Burlington, Canada), and centrifuged at 500× *g* for 20 min. Lymphocytes were collected from the interface of the liquid layers and filtered again through a 40-micron strainer. Lymphocytes were pooled in each group and stained with CD3-FITC, CD4-PerCP-Cy5.5, and CD8-BV711 antibodies (Becton Dickinson, Franklin Lakes, NJ, USA). All fluorophores were standardized with isotype controls (Becton Dickinson, Franklin Lakes, NJ, USA) and compensation using fluorescence minus one (FMO) standards. Additionally, a PE-conjugated IE1-specific tetramer (H-2L^d^, YPHFMPTNL) synthesized by the National Institutes of Health Tetramer Core Facility at Emory was used to determine MCMV-specific CD8+ T cells. Flow cytometry was conducted on a Fortessa X-20 machine (Becton Dickinson, Franklin Lakes, NJ, USA), and data were analyzed using FlowJo software (Becton Dickinson, Franklin Lakes, NJ, USA).

### 2.10. Statistics

All results were analyzed by using group mean and standard deviation or standard error of the mean, depending on the number of replicates conducted. Significance was determined with ANOVA with multiple comparisons using Tukey’s post hoc test or Kruskal–Wallis with Dunn’s multiple comparisons test. Additional analysis was conducted by using Mann–Whitney U test or Student’s *t* test to determine differences between individual groups. All statistics and figures were produced using Prism 8 software (Graph Pad, San Diego, CA, USA) Significance is shown as * *p* < 0.05; ** *p* < 0.001; *** *p* < 0.0001; and **** *p* < 0.00001.

## 3. Results

### 3.1. Characterization of MCMV-Associated Cardiac Dysfunction

Previously, we have shown that MCMV infection results in cardiac damage and dysfunction at 14 and 50 dpi. In order to further characterize the effects of MCMV infection within the heart, we questioned whether the damage and the dysfunction associated with MCMV infection of the heart were a result of viral modulation within the cell during the initial infection or whether they were consequences of viral replication. If viral replication is required, ganciclovir (GCV) treatment would significantly reduce MCMV replication within the heart and therefore limit cardiac dysfunction. For these studies, wild-type infected mice were either untreated or prophylactically treated with 50 mg/kg/day of GCV for 1 day and then as a therapeutic following infection for the duration of the study. At 1 day post-treatment, mice were infected with 1 × 10^6^ PFU of wild-type MCMV by i.p. inoculation. Consistent with previous studies, GCV treatment significantly reduced viral replication in the spleen, liver, lungs, and salivary glands at 3, 7, and 14 dpi [36]. Due to the limited replication previously shown within the heart, viral replication was evaluated only at 3 dpi [12,15,42]. As expected, GCV treatment resulted in significantly less replication within the heart compared to untreated animals (Figure S1). For this study, we anticipated that the inhibition of replication would result in less heart damage and dysfunction in the treated animals. However, echocardiography at 14 dpi demonstrated similar heart rates and left ventricular function in both groups (Figure S2). Generally, at 14 dpi, uninfected animals have heart rates of ~350–380 beats per minute, whereas both GCV-treated and untreated animals had heart rates of 424 and 435 beats per minute, respectively. These data indicate that although ganciclovir reduced the infectious MCMV load within the heart, it did not result in reduced dysfunction, suggesting that either early steps in viral infection or virus-induced host responses may be involved in this phenotype.

### 3.2. Acute Replication Kinetics and Viral DNA Load

Because ganciclovir did not reduce acute cardiac dysfunction, a viral mechanism prior to DNA replication, due to the initial infection or immediate–early gene expression or the induction of a host factor that initiates heart damage could account for the dysfunction. Since US28 is present on the virion and exerts its signaling effects upon virus entry, US28 may play a role in exacerbating chronic diseases. Likewise, the M33 vGCPR counterpart in MCMV is also expressed prior to DNA replication, and both proteins have been implicated in modulating smooth muscle migration.

**Figure 1:**
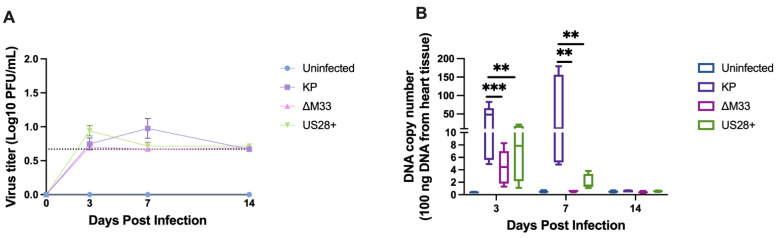
Wild-type and mutant MCMV acute infection within the heart. Animals were infected with 1 × 10^6^ PFU by i.p. inoculation. At 3, 7, and 14 dpi, hearts were analyzed for (**A**) viral replication by plaque assay on 3T3 fibroblasts. Studies were conducted in duplicate (*n* = 20) or (**B**) viral DNA load by qPCR using 100 ng DNA from heart tissue. DNA was quantified using a MCMV *early gene 1* (E1) plasmid. Studies were conducted once (*n* = 5). Group mean and standard error of the mean were calculated. Significance was determined by ANOVA and Tukey’s multiple comparisons test. ** *p* < 0.005 and *** *p* < 0.0005.

Thus, we next explored whether vGPCRs contribute to cardiovascular dysfunction [1,25,43,44]. Our previous study indicated a possible role for MCMV latency and reactivation in exacerbating cardiac dysfunction [15]. Given that a M33 knockout virus exhibits reduced levels of latency in the spleen and lungs, which leads to reduced reactivation [34], we sought to determine whether M33 also played a role in the establishment of a latent infection in the heart that led to dysfunction. To determine whether M33 contributed to heart dysfunction during MCMV infection, mice were infected with the wild-type MCMV strain KP, ΔM33, or US28+. To evaluate the mutant viruses for heart dysfunction, viral dissemination and viral replication within the heart were assessed following inoculation with 1 × 10^6^ PFU of KP, ΔM33, or US28+. At 3, 7, and 14 dpi, animals were sacrificed, and viral replication was evaluated using a plaque assay. As shown in Figure 1A, the wild-type MCMV KP strain disseminated to the heart and replicated for a limited period, which peaked at 7 dpi at 1 log PFU/mL tissue homogenate [12,15,42]. Both ΔM33 and US28+ were capable of dissemination to and replication within the heart; although a trend existed for both mutant MCMV viruses to replicate to lower levels than the wild type, this was not significant.

To further discern differences between the wild-type virus and the mutant viruses, the viral DNA levels were evaluated. DNA was extracted, and qPCR was conducted to determine the viral copy number within the heart. Interestingly, both ΔM33 and US28+ infections in the heart resulted in significantly lower levels of viral DNA at 3 and 7 dpi when compared with the KP-infected hearts (Figure 1B).

### 3.3. Acute Pathology

We previously showed that MCMV infection results in significant calcification and collagen deposition during acute infection, and we questioned whether vGPCR function contributes to this phenotype [15,39]. To further evaluate the mutant viruses during acute infection, hearts were assessed by estimated pathology scoring at 14 dpi and histology at 3, 7, and 14 dpi. When compared together by ANOVA, no significant differences were observed in the calcification of the epicardium between the groups (*p* = 0.13); however, when individually analyzed using a Mann–Whitney U test, increased calcification was observed in the US28+-infected animals (*p* = 0.02) compared with the uninfected controls (Figure S3). Though not significantly different, as in our previous paper, the KP-infected animals exhibited similar estimated pathology scores when individually analyzed by using a Mann–Whitney U test, suggesting that the overall trend had been maintained (*p* = 0.07).

**Figure 2:**
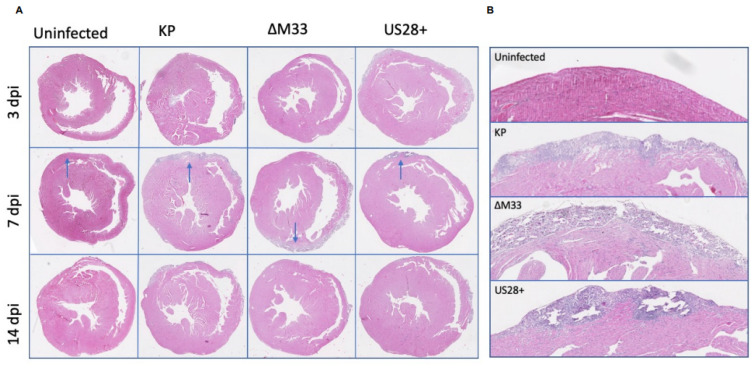
MCMV-induced immune cell infiltration during acute infection. Animals were uninfected or infected with 1 × 10^6^ PFU by i.p. inoculation. (**A**) At 3, 7, and 14 dpi, hearts were stained with hematoxylin and eosin to evaluate immune cell infiltration during acute infection. (**B**) Enlarged view of 7 dpi calcified zones (arrows). Images presented are a representative from each group. Study was conducted in duplicate (*n* = 2).

In addition to the calcification analysis, H and E staining was conducted to evaluate the damage and cellular infiltration within the heart during acute infection. This analysis revealed no differences between the groups at 3 and 14 dpi. However, at 7 dpi, MCMV infection caused mild myocarditis with lymphocyte infiltration, myofiber necrosis, and early stromal proliferation when compared with the uninfected controls (Figure 2). Owing to the small sample size, significant differences between the groups could not be determined by the pathologist; however, the US28+-infected animals exhibit increased damage in the calcified zone of the outer epicardium. While not significant, this result has been observed in previous preliminary studies and may suggest a role for US28 in promoting this damage within the heart.

### 3.4. Acute Cardiac Dysfunction

**Figure 3:**
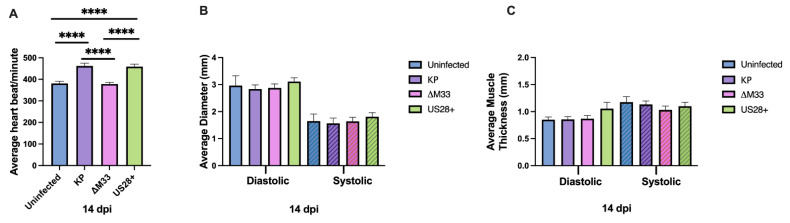
M33 and US28 promote acute tachycardia. Mice were uninfected or infected with 1 × 10^6^ PFU i.p. At 14 dpi, echocardiography was conducted: (**A**) heart rate, (**B**) left ventricular internal diameter, and (**C**) left ventricular posterior wall thickness were evaluated. Group mean and standard error of the mean were calculated. Significance was determined by ANOVA and Tukey’s multiple comparisons test. **** *p* = 0.0001. Studies were conducted in duplicate (*n* = 10).

As previously shown, MCMV-infected hearts demonstrated significant tachycardia compared with the uninfected control animals at 14 dpi; thus, we next explored whether vGPCRs contribute to MCMV-induced tachycardia [15]. Echocardiography at 14 dpi was conducted to evaluate cardiac function. Consistent with our previous studies, the KP and US28+ infections induced significant tachycardia (~462 beats per minute and ~459 beats per minute, respectively) compared with the uninfected controls (~381 beats per minute). Conversely, ΔM33-infected animals had significantly lower heart rates (~378 beats per minute), which were similar to the uninfected control mice (Figure 3). Interestingly, US28 complementation in the M33-deficient background restored the tachycardia such that it was comparable with that of wild-type virus. Despite the similar viral replication and pathology between viruses, this result suggests that M33 function promotes cardiac dysfunction during acute infection and that this can be rescued by US28. As shown in Figure 3B,C, and similar to our previous study, there were no significant differences between viruses or when compared with the uninfected animals in the functional parameters of the left ventricle at 14 dpi [15].

### 3.5. Cardiac Damage and Dysfunction during Latency

**Figure 4:**
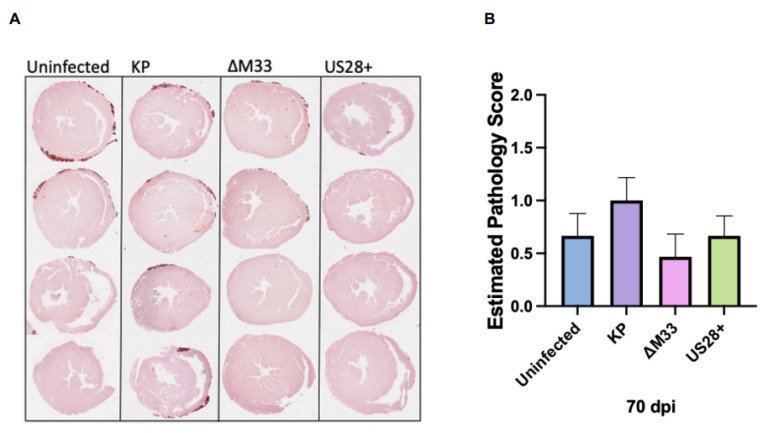
MCMV-induced calcification during latent infection. Animals were uninfected or infected with 1 × 10^6^ PFU by i.p. inoculation. At 70 dpi, (**A**) hearts were stained with Alizarin red solution to evaluate calcification in duplicate (*n* = 4). (**B**) Hearts were scored on the basis of the estimated pathology on the outer epicardium, as previously described. Studies were conducted in triplicate (*n* = 15).

We next investigated whether vGPCRs, and M33 in particular, contribute to long-term cardiac damage and dysfunction. Therefore, mice were again uninfected or infected with 1 × 10^6^ PFU of KP, ∆M33, or US28+ by i.p. inoculation and subsequently evaluated at 70 and 90 dpi. Similar to acute infection, at 70 dpi, there were no significant differences observed in calcification by Alizarin red staining or estimated pathology scoring (Figure 4). Uninfected BALB/c mice occasionally spontaneously develop calcification but MCMV exacerbates this phenotype [15,45]. However, at 70 dpi, we observed a biological trend approaching statistical significance (*p* = 0.07) when assessing the estimated pathology scores of the KP-infected hearts compared with the ∆M33-infected hearts that was not measured at 14 and 90 dpi between these groups (Figure S3). This suggests that M33 may contribute to promoting long-term calcification. At 90 dpi, the estimated pathology scores for all the infected groups were similar to those of the uninfected animals, suggesting that calcification due to MCMV infection had mostly resolved by this time point (Figure S3). Interestingly, the ∆M33-infected hearts exhibited the lowest scores among all the groups, suggesting that the trend observed at 70 dpi in the MCMV-infected animals had been maintained.

**Figure 5:**
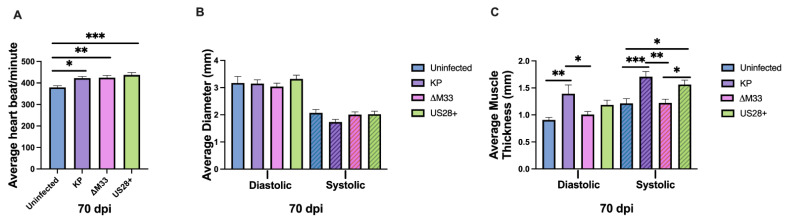
MCMV-induced cardiac dysfunction at 70 dpi. Mice were uninfected or infected with 1 × 10^6^ PFU i.p. At 70 dpi, echocardiography was conducted: (**A**) average heart rate per group, (**B**) average left ventricular diameter, and (**C**) average left ventricular posterior wall thickness were analyzed. Group mean and standard error of the mean were calculated. Significance was determined by ANOVA and Tukey’s multiple comparisons test. * *p* < 0.05, ** *p* < 0.005, *** *p* < 0.0005. Studies were conducted in triplicate (*n* = 15).

During latency, repeated viral reactivations may contribute to long-term dysfunction within the heart. Because M33 is required for efficient ex vivo reactivation, the use of a reactivation deficient virus presents an opportunity to explore whether repeated reactivation events contribute to the maintenance of altered heart function [15,34]. To test this, hearts were evaluated by echocardiography at 70 and 90 dpi. Unlike 14 dpi, at 70 dpi, there were no differences in the heart rates between the wild-type and mutant viruses; however, all three MCMV-infected groups demonstrated significantly higher heart rates than the uninfected control animals (Figure 5A). This suggests that during latency, vGPCRs are not essential to induce tachycardia and that the increased heart rate is likely due to other viral or host factors.

In addition to heart rate, several parameters of left ventricular function were also evaluated at 70 dpi. There were no significant differences observed for the internal diameter of the ventricle when compared together by ANOVA; however, when individually compared using the Mann–Whitney U test, the KP-infected animals exhibited a biological trend that was close to statistical significance (*p* = 0.053) for reduced internal diameter during systole compared with the uninfected hearts (Figure 5B). In addition, during both phases of heart beating, the KP and US28+-infected animals exhibited significantly increased posterior wall thickness (Figure 5C). Conversely, in the ∆M33-infected animals, the left ventricular posterior wall thickness was not significantly increased compared with the KP and US28+-infected mice and exhibited a similar thickness to that of the uninfected control animals. Individually, these two parameters are markers of cardiac hypertrophy and when shown together suggest exacerbated cardiac dysfunction. As the cardiac muscle expands, the posterior wall thickness increases, and this expansion can reduce the internal diameter of the ventricle, thus resulting in less-efficient hemodynamics. The significant increase in posterior wall thickness and the reduction in internal diameter observed in the KP-infected animals when compared with the uninfected controls together suggest that the dysfunction observed at 50 dpi, in our previous study, was exacerbated at 70 dpi [15]. In addition, Figure 5C suggests that M33 may contribute to the induction and exacerbation of hypertrophy within the heart.

**Figure 6:**
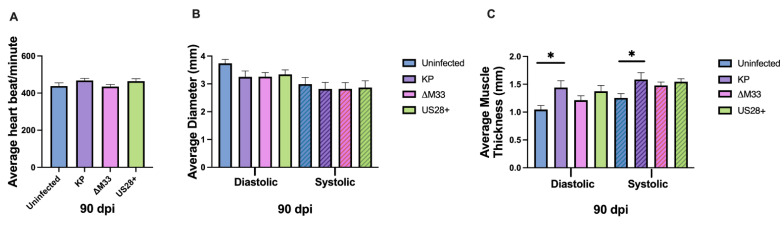
Cardiac function at 90 dpi. Mice were uninfected or infected with 1 × 10^6^ PFU i.p. At 90 dpi, echocardiography was conducted: (**A**) average heart rate per group, (**B**) average left ventricular diameter, (**C**) average left ventricular posterior wall thickness were analyzed. Group mean and standard error of the mean were calculated. Significance was determined by ANOVA and Tukey’s multiple comparisons test. * *p* < 0.05. Studies were conducted in triplicate (*n* = 15).

Echocardiography was also conducted at 90 dpi, and unlike 14 and 70 dpi, there were no significant differences in heart rate between the groups (Figure 6A). However, this was not because tachycardia resolved in the MCMV-infected animals but rather because the uninfected animals exhibited increased heart rates at this time point. In addition, we did not observe any significant differences in the average left ventricular internal diameter when analyzing all four groups using ANOVA (Figure 6B). However, when a Mann–Whitney U test was used to compare the KP-infected animals with the uninfected controls, we noted a potential biological trend (*p* = 0.077) in the diastolic phase of the heartbeat. Furthermore, at 90 dpi (Figure 6C), the left ventricular posterior wall thickness was no longer significantly different between the wild-type and mutant viruses, as was seen at 70 dpi (Figure 5C). However, the KP-infected animals maintained significantly thicker posterior walls (Figure 6C) compared with the uninfected control animals at 90 dpi. Overall, the differences between the wild-type viruses and the mutant viruses were largely resolved, suggesting that any potential role of vGPCRs in cardiac dysfunction observed at 70 dpi had dissipated by 90 dpi. While the KP-infected animals were trending toward reduced left ventricular internal diameters (Figure 6B) and retained the significantly thicker posterior walls (Figure 6C), the uninfected animals exhibited an altered cardiac phenotype compared with what was seen at previous time points, suggesting that the biological variation observed in older mice may diminish our ability to discern differences due to infection with age.

### 3.6. Modulation of Cardiac Gene Expression

**Figure 7:**
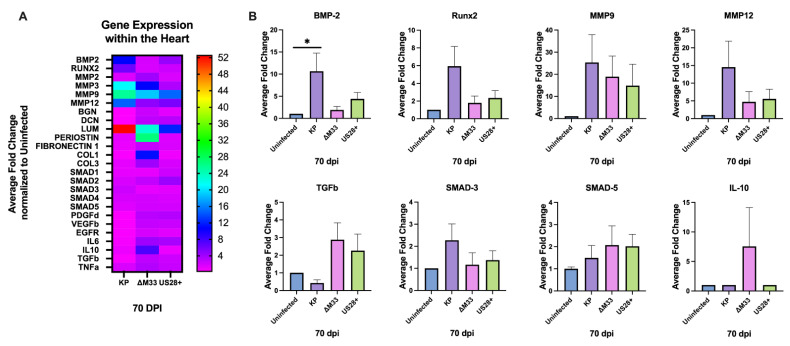
MCMV-induced upregulated gene expression at 70 dpi. Animals were uninfected or infected with 1 × 10^6^ PFU MCMV by i.p. inoculation. At 70 dpi, RNA was extracted from 0.1 g of tissue. Additionally, 100 ng of cDNA was synthesized per sample, and qPCR was conducted with a panel of cellular genes. Results are presented as (**A**) a heat map with color coding based on induced upregulated fold change or (**B**) a bar graph with the average fold change per group normalized to the uninfected samples. Two-log fold change was determined by using the cellular housekeeping gene *GAPDH.* Significance was determined by ANOVA and Tukey’s multiple comparisons test. * *p* < 0.05. This study was conducted once (*n* = 5).

Because vGPCRs are known to alter downstream signaling, which could contribute to modulations in the tissue microenvironment, we next evaluated whether there would be differences in cellular gene expression between the virus infection groups. At 70 dpi, an RT-PCR analysis was performed with a select pool of genes known to play a role in vascular growth, collagen deposition, and extracellular matrix (ECM) remodeling, as well as cytokines that have previously been shown to be altered from CMV infection [46,47]. MCMV infection of the heart at 70 dpi resulted in the upregulation of many genes compared with the uninfected control hearts (Figure 7A). Interestingly, hearts from the KP, ∆M33, and US28+ infected mice exhibited the upregulation of genes implicated in vascular smooth muscle motility, differentiation, and calcification. Of particular importance, both bone morphogenic protein 2 (BMP2) and runt-related transcription factor 2 (RUNX2), which are markers used to determine vascular calcification, were highly upregulated in the KP-infected hearts compared with the other groups [48,49,50]. Consistent with these findings, RT-PCR conducted at 90 dpi demonstrated an upregulation in BMP2 and RUNX2 in the KP- and US28+-infected mice but not in the uninfected mice or the ∆M33-infected animals (Figure S4). This supports the biological trend observed in Figure 4 and suggests that M33 plays a role, and that US28 might also play a role, in the induction and exacerbation of calcification within the heart, as shown at the transcriptional level.

This preliminary snapshot of host genes also demonstrates that matrix metalloproteinases (MMPs) were highly upregulated in the MCMV-infected hearts when compared with the uninfected control animals. Although all three viruses were capable of inducing MMPs to some degree, the KP-infected animals demonstrated the highest induction, suggesting that in the absence of M33, MMPs and other ECM genes are subject to less modulation. However, this reduced modulation greatly varied between the genes; for example, MMP9 exhibited similar induction, whereas MMP12 was differentially expressed between the KP-infect hearts and the ∆M33-infected hearts. Because both MMP9 and MMP12 have been shown to be important in ECM remodeling, additional studies are needed to further understand this phenotype [51,52,53].

Conversely, SMAD signaling, growth factors, and most of the immune markers evaluated were not upregulated within the heart of the MCMV-infected animals. This may be due to the time point analyzed because the hearts are latently infected; however, given the exacerbated damage, we expected that there would have been an upregulation of these markers when compared with the uninfected hearts. Interestingly, there was an upregulation of IL6, TGFβ, and IL10 mRNA expression in the ∆M33-infected hearts that was not detected in the other groups. This suggests a role for M33 in the modulation of cytokine production in the heart. Recently, similar results have been found in aortic allografts at 30 dpi, where IL10 expression was significantly upregulated in the absence of M33 when compared with the wild-type MCMV-infected grafts [26]. Together, Figure 4 and Figure 5 demonstrate that MCMV infection causes long-term phenotypic changes in the heart and that M33 may play a role in this modulation, as demonstrated by the reduced calcification and altered gene expression observed in the absence of the M33 protein.

### 3.7. MCMV Reactivation and Immune Memory

Previously, we demonstrated that MCMV readily reactivates from the heart using an explant reactivation assay and we hypothesized that repeated reactivations may induce changes in the microenvironment of the heart, which over time could exacerbate cardiovascular diseases [15]. The ∆M33 virus is less efficient in reactivation from the spleen and lungs when compared with the wild-type MCMV [34]. If this deficiency also occurs within the heart, then this may contribute to the phenotypic differences between viruses shown at 70 dpi (Figure 5). In order to test this, the explant reactivation assay was conducted to examine ∆M33 and US28+ reactivation capacities within the heart. This assay allows tissues to be cultured ex vivo to evaluate their latency status and capacity for reactivation. Weekly supernatant is assessed by using a plaque assay to demonstrate reactivation. If there are no plaques at 7 days post explantation, then the tissue is considered latently infected at the time of explant. Plaques found in the following weeks (post explant) demonstrate MCMV reactivations within the explanted tissue [34].

**Figure 8:**
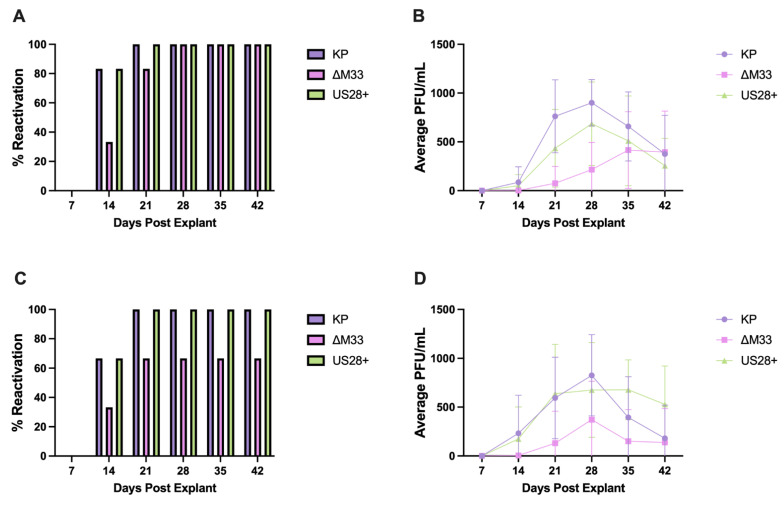
Ex vivo reactivation of MCMV from the heart. Animals were infected with 1x10^6^ PFU by i.p. At 70 dpi (**A**,**B**) and 90 dpi (**C**,**D**), hearts are cultured ex vivo for 6 weeks to evaluate MCMV reactivation. (**A**,**C**) The percentage of animals per group whose hearts reactivated the virus was determined by using a plaque assay. (**B**,**D**) The average PFU/mL supernatant from each group was determined by using a plaque assay. Studies were conducted in duplicate (*n* = 6) at 70 dpi and once (*n* = 3) at 90 dpi.

As shown in Figure 8, all three viruses reactivated from latency at 70 and 90 dpi. However, similar to what has previously been shown in other tissues, ∆M33-infected hearts demonstrate a deficiency in the percentage of hearts that reactivate and in the quantity of progeny virus produced. This suggests that the M33 protein is not required for reactivation within the heart but that it plays a role in promoting reactivation and viral replication following reactivation or that the ∆M33 latent load is reduced. Thus, fewer latent genomes can reactivate from latency. Interestingly, reactivation within the spleen (Figure S5) results in reduced progeny virus production in all three groups compared with the heart, which could be caused by differences in the latent cell population, explanted culture conditions, or immune control within the spleen than what occurs within the heart. In addition, in the absence of M33, the US28 protein restored the ability of the virus to reactivate and produce progeny in the heart, and to a lesser degree in the spleen, suggesting that US28 complements M33 function during latency or reactivation from latency in a tissue-specific manner [27].

Long-term CMV infection drastically inflates the CMV-specific CD8 T-cell populations in HCMV-seropositive patients and MCMV-infected mice [54,55,56]. Based on the reduced damage, dysfunction, and reactivation observed in ∆M33-infected hearts, we next evaluated the CD8 T-cell populations between the groups to test whether reduced in vivo ∆M33 reactivation resulted in differences in MCMV-specific CD8 T cells in ∆M33-infected hearts. At 90 dpi, five hearts per group were pooled and analyzed through flow cytometry to evaluate the IE1-specific CD8 T-cell populations between viruses. T cells were detected in roughly the same numbers between the groups; however, the percentage of IE1-specific CD8 T cells was nearly double in KP and US28+-infected hearts compared with ∆M33-infected hearts (Figure S6). Although this study is preliminary, when combined with the explant reactivation data, these results may indicate that increased IE1-specific CD8+ T cells in the wild-type and US28-infected hearts reflect the response to IE1 gene expression during reactivation from latency. These results support the idea that in the absence of M33, reactivation events may occur less often or less replication occurs after in vivo reactivation and leads to less damage and dysfunction within the heart [57].

## 4. Discussion

HCMV seropositivity is associated with many cardiovascular diseases, including myocarditis, transplant vasculopathy, vascular sclerosis, and hypertension [3,4,5,6,7,8,9]. We have previously shown that MCMV infection of the heart results in phenotypic and functional changes during acute and latent infection [15]. In order to further characterize the effects of MCMV infection within the heart, we first evaluated whether ganciclovir treatment could diminish the damage observed during acute infection. Interestingly, MCMV-induced cardiac dysfunction did not require virus replication, because treating mice with ganciclovir did not significantly reduce tachycardia at 14 dpi. Another potential determinant of cardiac dysfunction could result from the initial infection events, such as the entry or induction of signaling prior to viral replication beginning. This led us to explore whether two virally encoded GPCRs, M33 and US28, contribute to cardiac dysfunction signaling cascades. Both US28 and M33 are known to modulate many signaling pathways responsible for cellular remodeling, angiogenesis, proliferation, and migration [28,34,43,58,59,60,61]. Furthermore, US28 has been implicated in the viral modulation that exacerbates the development of chronic diseases [1,25,26]. Additionally, M33 plays a role in MCMV latency and reactivation [34]. Thus, we took advantage of available wild-type parent and mutant viruses, specifically ∆M33 and US28+, to evaluate the role of vGPCRs and whether viral reactivation may exacerbate heart dysfunction [21,35].

Similar to some of the other tissues, both ∆M33 and US28+ viruses disseminated to and replicated in the heart to comparable levels as wild-type KP during acute infection [15,34]. No significant differences in calcification, pathology, or myocarditis were detected at 3 and 14 dpi between the groups. This suggested that despite M33’s role in cell signaling, it did not significantly contribute to cardiac damage during acute infection. Conversely, echocardiography at 14 dpi demonstrated significantly increased heart rate in the KP-infected and US28+-infected animals compared with the ∆M33-infected animals and the uninfected controls (Figure 3). This suggests that M33 plays a role in the induction of tachycardia during acute infection and that perhaps US28 plays a similar role during HCMV infection.

As we have previously shown, latent MCMV infection exacerbates cardiac damage and dysfunction [15]. To determine whether viral latency and in vivo reactivation contributed to the heart phenotype, we took advantage of the ∆M33 mutant virus, which is deficient in latency and reactivation, to answer this question. If reactivation from latency contributes to long-term heart damage, we predicted that there would be reduced cardiac injury in ∆M33-infected mice. At 70 dpi, the KP-infected animals had somewhat greater calcification on the epicardium of the heart than the ∆M33-infected animals (Figure 4). This finding is supported by our RT-PCR results at both 70 and 90 dpi, where the calcification markers BMP2 and RUNX2 were upregulated in the KP- but not the ∆M33-infected hearts (Figure 7). The BMP2 and RUNX2 genes are involved in the transdifferentiation of smooth muscle cells into an osteoblastic phenotype known to secrete calcium vesicles into the extracellular spaces of soft tissues, including the vessels [49,50,62]. Together, these findings indicate that M33 may induce and exacerbate calcification during latency or reactivation events through the upregulation of factors that control calcium levels within the heart. Indeed, we recently demonstrated that infection alone is sufficient to induce calcification in murine aortic smooth muscle cells [39].

In addition to calcification, the KP-infected animals had an increased expression of MMPs, which function in matrix degradation during ECM remodeling [50,63]. MMPs are important modulators of cardiovascular dysfunction that are upregulated within the aorta of patients with calcified aortic valve disease. The removal of MMP9 or MMP12 in ApoE-/- mice protects these animals from plaque development and rupture [51,53,64]. Both HCMV and MCMV upregulate MMPs during lytic infection in cell cultures, but to the best of our knowledge, this is the first study to show upregulation in the heart during latent MCMV infection and show that it most likely contributes to long-term cardiac dysfunction [46,47,60,65,66,67].

To our surprise, the KP-infected mice did not exhibit an upregulation of signaling and immune markers compared with the uninfected animals (Figure 7), but our analysis was conducted only during latency, because this was the time when we measured the highest degree of dysfunction. A previous study on MCMV infection in the lungs found TGFβ expression during latency that was associated with fibrosis of the tissue [68]. Given that TGFβ is known to induce fibrosis and calcification via SMAD signaling, we expected both TGFβ and SMADs to be upregulated, but we did not observe this [48]. However, a role for TGFβ in the development of fibrosis in the heart at earlier times cannot be ruled out. Interestingly, the only upregulated immune markers were in the ∆M33-infected hearts, where the mRNA expression of IL6, TGFβ, and IL10 were upregulated in the absence of M33. This result raises the possibility that M33 regulates aspects of the inflammatory response that could contribute to heart dysfunction. Although there were no longer significant differences in heart rate or left ventricular internal diameter values between the viruses (Figure 5), there were reduced internal diameter values during systole in the KP-infected hearts, suggesting hypertrophy in the wild-type MCMV-infected animals. This was confirmed with left ventricular posterior wall thickness measurements, which demonstrated that the KP and US28+-infected animals had significant hypertrophy when compared with the uninfected animals and the ∆M33-infected animals. These results suggest that over time, in the absence of M33, less damage is occurring, and therefore, less compensatory hypertrophy is needed to maintain cardiac output [15,69,70]. Interestingly, at 90 dpi, the functional differences observed between the viruses at 70 dpi had resolved. The KP-infected hearts, though, still exhibited significant hypertrophy when compared with the uninfected mice, but this was no longer true when compared with the ∆M33-infected animals (Figure 6).

From our studies, we propose that CMV replication causes phenotypic changes within the microenvironment of the heart following primary infection. CMV establishes latency within the heart and most likely further exacerbates damage and dysfunction through frequent reactivation events which produce progeny virus and the induction of inflammation. Because ∆M33 is deficient in reactivation from the spleen and lungs, it is possible that the reduced dysfunction observed at 70 dpi in the ∆M33-infected hearts may be associated with reduced in vivo reactivation [34]. We show that ex vivo ∆M33 reactivation from the heart is less efficient than KP, demonstrated by both the reduction in the percentage of reactivation and the lower quantity of progeny virus production. For either case, decreased reactivation and the reduction in the infectious virus could stimulate less damage in the heart. A decrease in the reactivating virus could also be expected to result in decreased T-cell infiltration and/or response in the heart. Both HCMV and MCMV induce a clonal expansion of epitope-specific CD8+ T cells, known as memory inflation [54,55]. Although preliminary, our flow cytometry analysis of MCMV IE1-specific CD8 T cells indicates reduced numbers of virus-specific T cells in the latent hearts of ∆M33-infected mice. The absolute numbers of the CD8+ T cells were low in all hearts, yet we found approximately double the percentage of IE1-specific CD8 T cells in mice infected with KP or US28+ compared with that of ∆M33-infected mice. Thus, in the absence of M33, fewer T cells may be stimulated to control reactivating viral replication in the heart (Figure S6). Studies to understand the T-cell response to wild-type MCMV and ∆M33 were recently conducted, and they indicate that there is reduced T-cell surveillance in the M33-deficient virus-infected mice [57]. Further studies are needed to understand the T-cell response in the heart and whether decreased reactivation results in decreased T-cell surveillance in the heart.

## 5. Conclusions

HCMV infection is associated with cardiovascular disease [3,4,5,6,7,8,9]. Using the MCMV-infection model, our results demonstrate that MCMV infection results in cardiac damage and dysfunction during acute and latent infection and that US28 and M33 contribute to cardiac dysfunction to some degree. In the absence of M33, heart rate reduction occurs during acute infection. During latency, calcification, ECM remodeling, heart rate, and left ventricular dysfunction occur significantly less often in ∆M33-infected animals. Additional studies are necessary to determine the mechanisms involved in MCMV-induced heart dysfunction and how this translates to long-term HCMV infection and cardiovascular diseases. Importantly, because US28 expression restores cardiac damage, US28 may play a similar role in HCMV-infected hearts and may represent a potential therapeutic target for reducing damage and dysfunction within the vascular system of HCMV-seropositive individuals.

## Data Availability

Data are available upon request to the corresponding author.

## Supplementary Materials

The following supporting information can be downloaded at https://www.mdpi.com/article/10.3390/v15030711/s1. Table S1: Primer list used for cellular gene expression studies (Figure 7 and Figure S4). Figure S1: Ganciclovir treatment significantly reduces MCMV kinetics in the mouse. Figure S2: The effects of ganciclovir treatment in cardiac function at 14 dpi. Figure S3: MCMV infection does not induce significant calcification at 14 or 90 dpi. Figure S4: MCMV-induced calcification at 90 dpi. Figure S5: Ex vivo reactivation of MCMV from the spleen. Figure S6: Percentage of MCMV-specific IE1 tetramer-positive CD8 T cells from the heart.
