# Supplementary material for: The Viral G-Protein-Coupled Receptor Homologs M33 and US28 Promote Cardiac Dysfunction during Murine Cytomegalovirus Infection
Source: Viruses. 2023 Mar 9;15(3):711. doi: 10.3390/v15030711 (PMC10054303; doi:10.3390/v15030711)
Supplement: Supplementary file 1 [file viruses-15-00711-s001.zip › Supplementary Figures.pdf]

Supplementary Figures

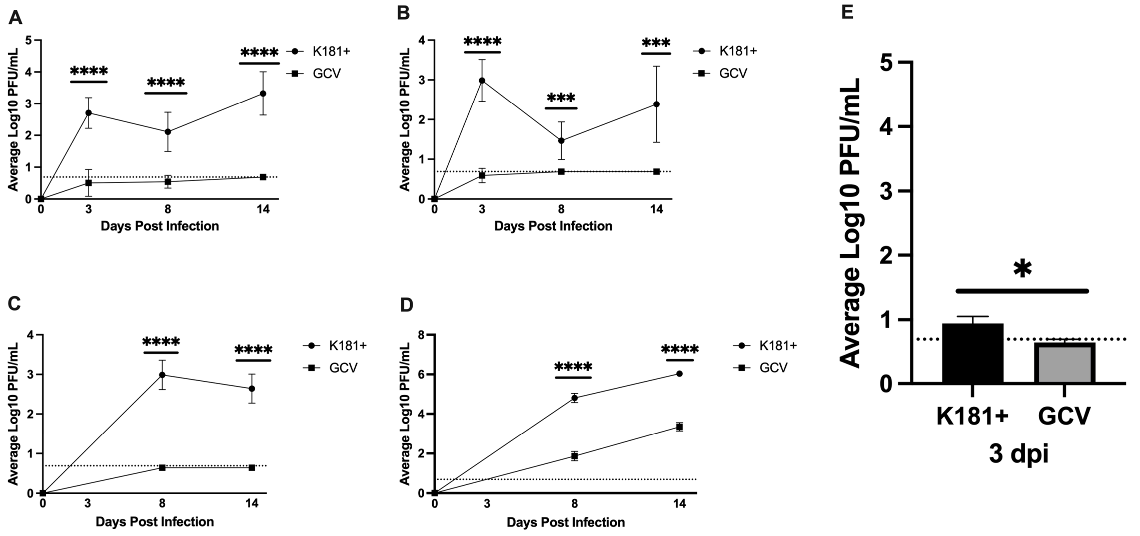

Figure S1. Ganciclovir treatment significantly reduces MCMV kinetics in the mouse. Mice were treated with 50 mg/kg GCV daily and then inoculated i.p. with 1x10^6 PFU of MCMV. At 3, 8, and 14 dpi, viral kinetics were evaluated in the (A) spleen, (B) liver, (C) lung, and (D) salivary gland by plaque assay. (E) MCMV replication of the heart was evaluated at 3 dpi. Group mean and standard error of the mean were calculated, and significance was determined by Student's T test. The study was conducted once (N=8).

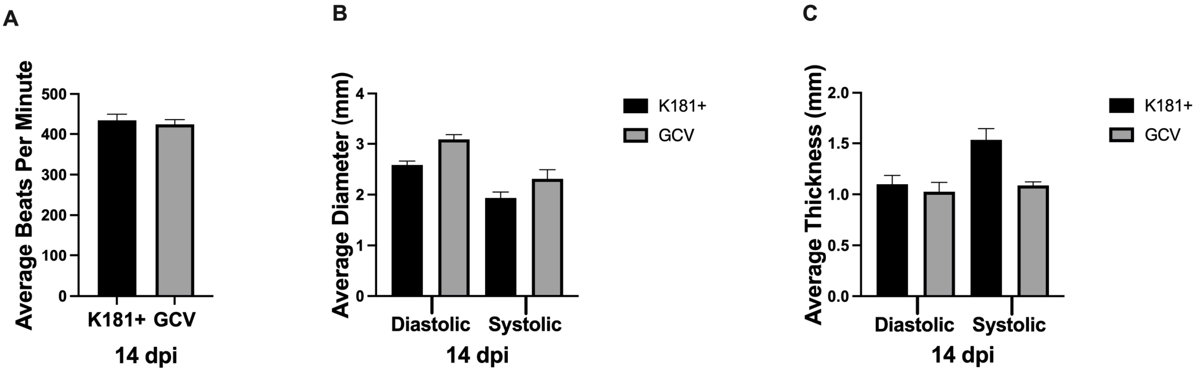

Figure S2. The effects of ganciclovir treatment in cardiac function at 14 dpi. Mice were treated with 50 mg/kg GCV daily and then inoculated i.p. with 1x10^6 PFU of MCMV. At 14 dpi, echocardiography was conducted and (A) heart rate, (B) Left ventricular internal diameter, and (C) left ventricular posterior wall thickness were evaluated. Group mean and standard deviation was calculated, and significance was determined by Student's T test. The study was conducted once (N=8).

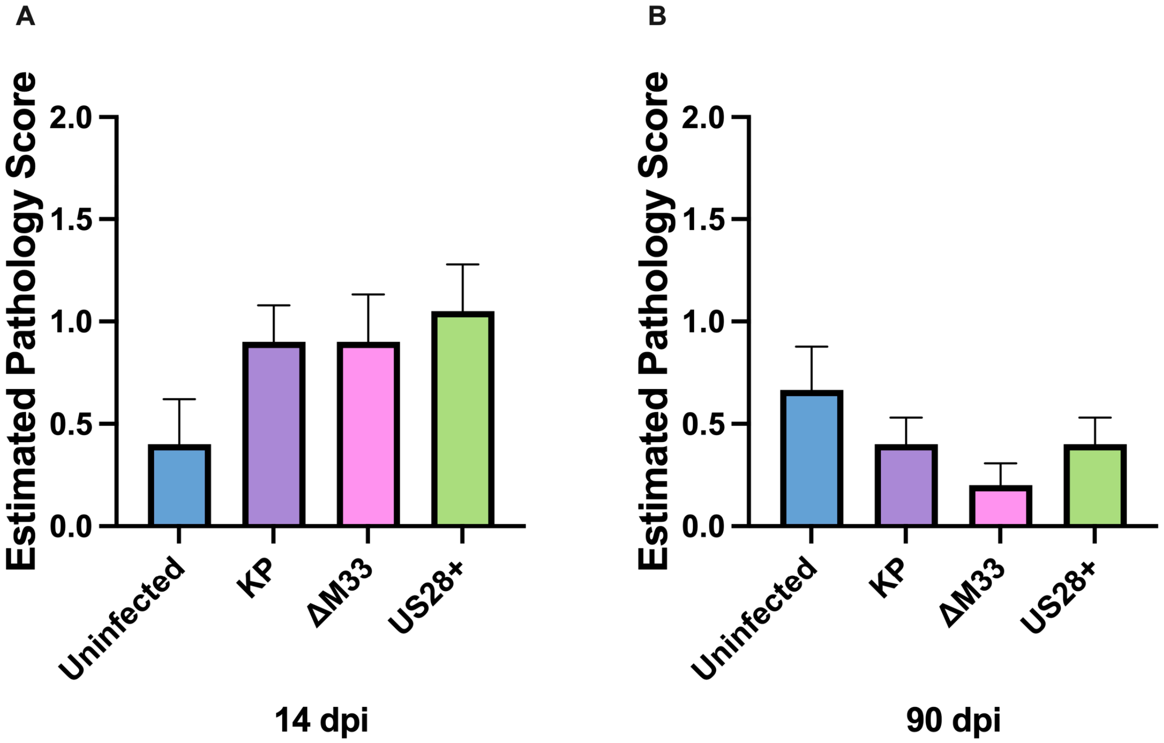

Figure S3. MCMV-infection does not induce significant calcification at 14 and 90 dpi. Animals were uninfected or infected with 1x10^6 PFU MCMV by i.p. inoculation. At (A) 14 dpi and (B) hearts were scored based on the estimated pathology on the outer epicardium as previously described. Studies were conducted in duplicate (N=20) at 14 dpi and in triplicate (N=15) at 90 dpi.

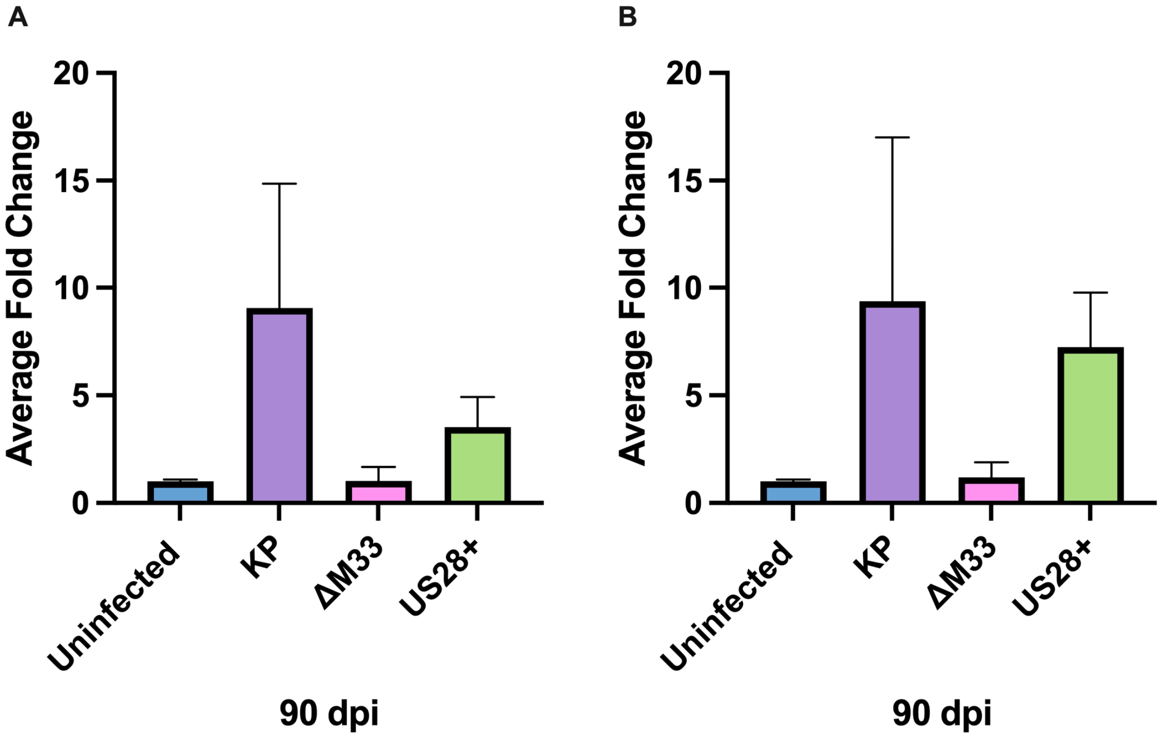

Figure S4. MCMV-induced calcification at 90 dpi. Animals were uninfected or infected with 1x10^6 PFU MCMV by i.p. inoculation. At 90 dpi, RNA was extracted from 0.1 gram of tissue, cDNA was synthesized, and qPCR was conducted with (A) BMP2 or (B) RUNX2 specific primers. Results are presented as the average fold change per group which was normalized to the uninfected samples. Two log fold change was determined using the cellular housekeeping gene *GAPDH.* qPCR analysis was conducted once (N=5).

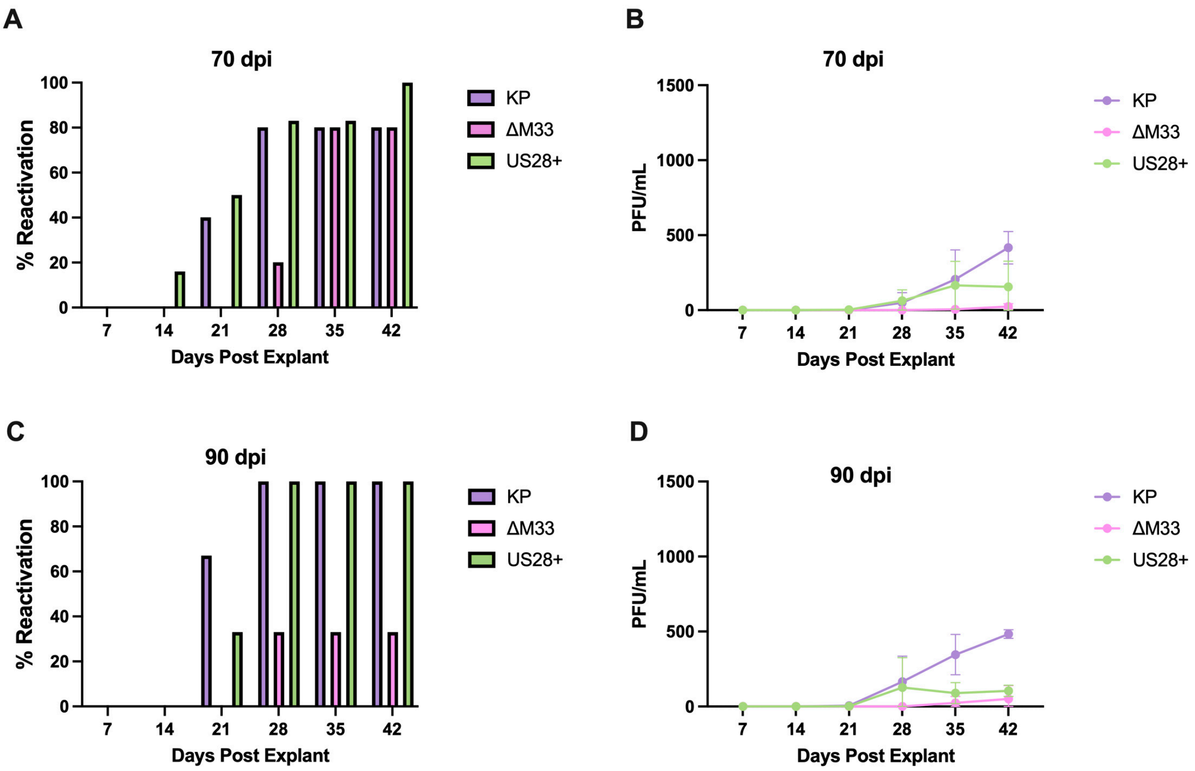

Figure S5. Ex vivo reactivation of MCMV from the spleen. Animals were inoculated with $1x10^6$ PFU by i.p. inoculation. At 70 dpi (A & B) and 90 dpi (C & D), spleens were cultured ex vivo for 6 weeks to evaluate MCMV reactivation. (A & C) The percentage of animals per group whose spleens reactivated determined by plaque assay, (B & D) the average PFU/mL supernatant from each group determined by plaque assay. Studies were conducted in duplicate (N=5-6) at 70 dpi and once (N=3) at 90 dpi.

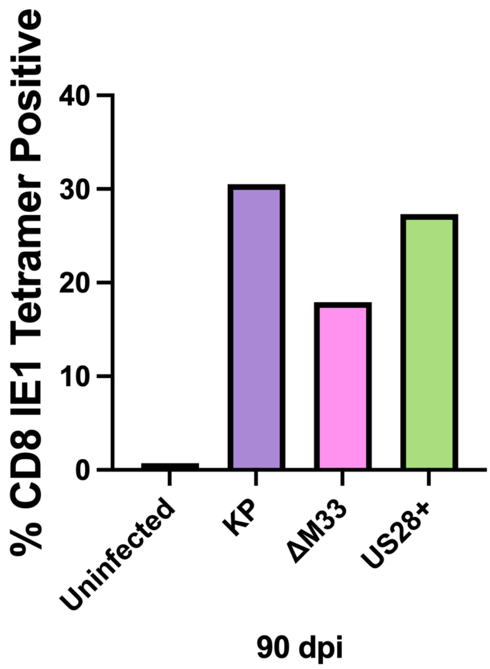

Figure S6. Percentage of MCMV-specific IE1 Tetramer positive CD8 T cells from the heart. Animals were inoculated i.p. with 1x10^6 PFU. At 90 dpi, 5 hearts per group were homogenized, pooled, and the cells were stained with MCMV-specific IE1 tetramer and flow cytometry was conducted. Cells were gated by CD3, CD4, CD8, and IE1 tetramer. The percentage of CD8 T cells specific for MCMV IE1 tetramer was calculated using flow jo software.
