# Supplementary material for: The Viral G-Protein-Coupled Receptor Homologs M33 and US28 Promote Cardiac Dysfunction during Murine Cytomegalovirus Infection
Source: Viruses. 2023 Mar 9;15(3):711. doi: 10.3390/v15030711 (PMC10054303; doi:10.3390/v15030711)
Supplement: Supplementary file 1 [file viruses-15-00711-s001.zip › Supplementary Table S1.pdf]

Table S1. Primer list used for cellular gene expression studies (Figure 7 and Figure S5).

BMP2	F 5'-AGTAGTTTCCAGCACCGAATTA-3'	R 5'-TTCACTAACCTGGTGTCCAATAG-3'
RUNX2	F 5'-TGGCTTGGGTTTCAGGTTAG-3'	R 5'-GGTTTCTTAGGGTCTTGGAGTG-3'
MMP2	F 5'-CTGGAATGCCATCCCTGATAA-3'	R 5'-GGTTCTCCAGCTTCAGGTAATAA-3'
MMP3	F 5'-GGATCTTCGCAGTTGGAATTTG-3'	R 5'-GATGCCTTCCTTGGATCTCTT-3'
MMP9	F 5'-TGCACTGGGCTTAGATCATTC-3'	R 5'-TGCCGTCTATGTCGTCTTTATTC-3'
MMP12	F 5'-CTGGAGGTATGATGTGAGGCAG-3'	R 5'-CTGGGAAGTGTGTGGAAATCAG-3'
Biglycan	F 5'-ATTGCCCTACCCAGAAACTTGAC-3'	R 5'-GCAGAGTATGAACCCTTTCCTG-3'
Decorin	F 5'-TGATGCACCCAGCCTGAAAG-3'	R 5'-TCCATAACGGTGATGCTGTTGAA-3'
Lumican	F 5'-TCGAGCTTGATCTCTCCTAT-3'	R 5'-TGGTCCCAGGTCTTACAGAA-3'
Periostin	F 5'-GATAAAATACATCCAAATCAAGTTTGTTCG-3'	R 5'-AAACTCTGTGGTCTGGCCTCTGGG-3'
Fibronectin 1	F 5'-GTGCTTCATGCCGCTAGAT-3'	R 5'-GTGTGGATTGACCTTGGTAGAG-3'
Collagen Type 1	F 5'-TTCTAGTTCCTGGGCCTATCT-3'	R 5'-GATGCAGGACAGACCAAGAG-3'
Collagen Type 3	F 5'-GGGAAAGGAGTCCACCTTTATT-3'	R 5'-GAATCTGTCCACCAGTGCTTAC-3'
SMAD1	F 5'-GGGAAAGGAGTCCACCTTTATT-3'	R 5'-GAAAGCCGTGGTGGTAGTT-3'
SMAD2	F 5'-GCTGAGTGCCTAAGTGATG-3'	R 5'-TACAGCCTGGTGGGATCTTA-3'
SMAD3	F 5'-TTCCATTCCCGAGAACACTAA-3'	R 5'-TCACTGGTTTCTCCATCTTCAC-3'
SMAD4	F 5'-CCAGGATGGACTATGAAAT-3'	R 5'-GCAGCAAACACATCTCTCAAC-3'
SMAD5	F 5'-GATTGTTGGGGAAACAAG-3'	R 5'-CTCCATAGCACCCTTCTTCTTC-3'
PDGFd	F 5'-CGCAGAGAGCATCCATCAA-3'	R 5'-GTCACCTGAATGTTCTCCTCTC-3'
VEGFb	F 5'-TCTGAGCATGGAACTCATGG-3'	R 5'-TCTGCATTCACATTGGCTGT-3'
EGFR	F 5'-AGAGCGCCTTCCACAGCCAC-3'	R 5'-ACTCTCGGAACTTTGGGCGG-3'
IL6	F 5'-ACACATGTTCTCTGGGAAATCGT-3'	R 5'-AAGTGCATCATCGTTGTTCATACA-3'
IL10	F 5'-ATGCTGCCTGCTCTTACTGACTG-3'	R 5'-CCCAAGTAACCCTTAAAGTCCTGC-3'
TGFb	F 5'-ATGCTAAAGAGGTCACCCGC-3'	R 5'-TGCTTCCCGAATGTCTGACG-3'
TNFa	F 5'-CCACGCTCTTCTGTCTACTGA-3'	R 5'-GCTACAGGCTTGTCACTCG-3'
GAPDH	F 5'-CTCACAATTTCCATCCCAGAC-3'	R 5'-TTTTTGGGTGCAGCGAAC-3'
